# Effectiveness of Strengthening Exercises for the Elderly with Low Back Pain to Improve Symptoms and Functions: A Systematic Review

**DOI:** 10.1155/2016/3230427

**Published:** 2016-05-16

**Authors:** Nor Azizah Ishak, Zarina Zahari, Maria Justine

**Affiliations:** Department of Physiotherapy, Faculty of Health Sciences, Universiti Teknologi MARA, 42300 Puncak Alam, Selangor, Malaysia

## Abstract

*Objective*. To determine the effect of strengthening exercises for older people with low back pain (LBP).* Methods*. This study is a systematic review of experimental study which evaluated the evidence regarding exercises for older people with LBP by using EBSCO Academic Search Premier, EBSCO EconLit, Science Direct, PUBMED, and PEDro from 2006 to 2016. Search strategy for each database was conducted by using keywords such as “low back pain”, “older people”, and “strengthening exercise”. Boolean operators were used to combine keywords and manual exclusion was conducted to verify studies which met the inclusion criteria. The articles reviewed were evaluated and critically appraised by using PEDro scale and SPSS version 20 was used to analyze the data.* Results*. Three articles were found regarding strengthening exercise for older people with LBP whereas one study was conducted on multicomponent exercise. The mean, standard deviation, and variance of the PEDro score of all the studies were 5.67, 2.33, and 1.528, respectively. Overall, the qualities of all studies reviewed were fair. Two articles showed significant results when compared to control group (*p* < 0.05).* Conclusions*. Strengthening exercise is a beneficial treatment for older people with LBP in reducing pain intensity, disability, and improved functional performances.

## 1. Introduction

Low back pain (LBP) is a major musculoskeletal problem experienced by many individuals at some period in their lives [1]. LBP is a common complaint associated with functional limitations and disability among elderly individuals. As such, the rehabilitation for LBP has been recommended to manage and reduce the prevalence of this condition among the elderly. The effective treatment for LBP has been extensively investigated to improve their physical functions and quality of life and to reduce burden to families, societies, and countries.

The recommended physiotherapy management for LBP comprises a wide range of treatment strategies, including electrotherapy, manual therapy, cognitive behavioral therapy, and exercise [2–6]. Exercise has also been widely applied by physiotherapists in clinical settings to treat LBP [7] and to encourage self-care management, which emphasizes a patient's active participation and efforts to manage LBP [8]. Moreover, exercise therapy focuses on the prescription of muscular contraction and body movement to improve overall health [9]. Therefore, exercise may protect and improve mobility and function, which help maintain the body functions of the elderly.

Several exercise types, including pilates, stretching, aerobics, and strengthening exercise, have been addressed among the elderly with LBP. However, strengthening exercises have been a major concern among clinicians and researchers because this type of exercise has been included in their exercise program because it improves the muscle strength of the elderly with LBP. Shnayderman and Katz-leurer [10] revealed that strengthening exercise is more effective than aerobic exercise for chronic LBP. Hyoung [11] also reported that exercise for the lower back of elderly women with chronic LBP strengthens the lumbar muscle extensor. The pain and disability scores of women with LBP are significantly lower than those of the control group. As such, strengthening exercises have been widely recommended for subjects with LBP because of their positive effects.

Since studies were conducted on exercises for the treatment of LBP, several systematic reviews have been performed to critically analyze and review the relevance and effectiveness of exercise therapy for subjects with LBP. Van Middelkoop et al. [12] indicated that exercise therapy decreases pain intensity, alleviates disability, and improves physical functions for a long period, such as 12-month follow-up. Meta-analyses have also been conducted on the effectiveness of exercise as LBP treatment [5, 9, 10]. Slade and Keating [13] systematically reviewed trunk strengthening exercise and revealed that this exercise can alleviate pain and improve functions more effectively than aerobics and other exercises do. The effects of treatment are enhanced if intensity and motivation strategies are included. Taylor et al. [14] reported that the progressive resistance exercise is effective and safe for many patients experiencing muscle force deficiency and pain-related problems. Taylor et al. [14] also found that exercise improves the ability of patients to perform daily life activities. However, studies have been conducted among the general population because the age group eligible for this is not stated. Studies have yet to investigate elderly people with LBP because subjects belong to the general population. The effectiveness of strengthening exercises and their suitability as an intervention to reduce the pain intensity caused by LBP among the elderly remain unclear, although strengthening exercises have been applied in clinical practice. Therefore, studies should be conducted to verify whether strengthening exercises are effective for the elderly before such exercises are prescribed. This study aimed to determine whether strengthening exercises can reduce the symptoms of LBP among the elderly.

## 2. Methods

### 2.1. Research Strategies

Literature search was conducted through several steps. First, the objective of this study was defined with population, intervention, comparison, and outcome (PICO) techniques. These techniques were determined to establish the eligibility criteria for this study as follows.

 Population: older people/age 60 years and above, presenting with LBP.
 Intervention: strengthening exercise.
 Comparison: pre- and postintervention.
 Outcome: pain measurements (numerical rating scale).

**Figure 1:**
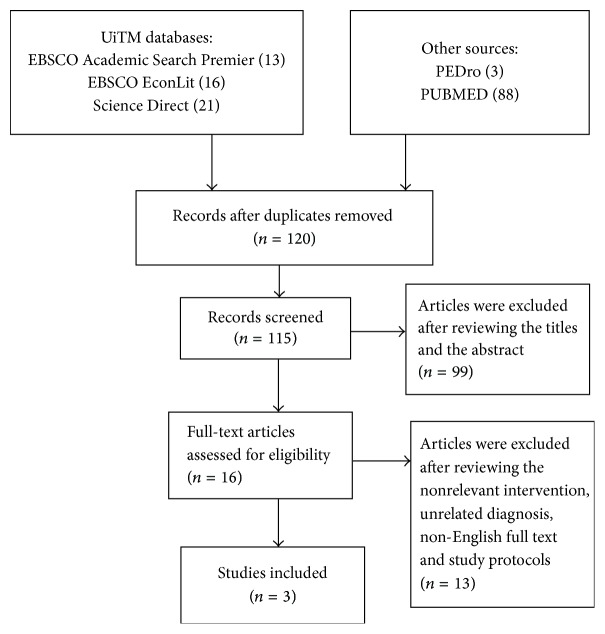
PRISMA flow diagram of search strategies.

Second, our literature search was performed between 2006 and 2016 through the following databases: EBSCO Academic Search Premier, EBSCO EconLit, Science Direct, PUBMED, and PEDro. The search was limited to 10 years to obtain current articles regarding the implementation of strengthening exercises for elderly people with LBP. Each database was searched by using the following keywords: “low back pain”, “elderly”, and “strengthening exercises” (see Table 1). Boolean operators were used to combine two or more keywords. Manual exclusion was conducted to verify whether studies satisfied the inclusion criteria. Studies were selected by using search engines on the basis of title, study design, methodologies, intervention, and population. Experimental studies on back exercises prescribed for elderly people were chosen on the basis of the inclusion criteria (Figure 1). Sixteen articles on exercises for LBP were initially found [13–28]. However, only three studies were included after the standardized pain measurement parameter was used. The study types included in this research were experimental studies, randomized controlled trials (RCTs), and observational cohort. All of the studies were ranked on the basis of the hierarchy levels of evidence in accordance with the National Health and Medical Research Council (NHMRC) [29]. The Preferred Reporting Items Systematic Reviews and Meta-Analysis (PRISMA) statement was followed.

### 2.2. Study Selection

#### 2.2.1. Inclusion Criteria

Studies were included when the following criteria were satisfied: they recruited elderly people or individuals aged 60 and above with LBP diagnosed as acute, subacute, or chronic; people with specific or nonspecific LBP and osteoporosis at the lumbar and vertebral fracture (lumbar region); and people who included the strengthening exercises in their health programs. Studies were also selected when they were characterized as full-text language articles and those that used outcome measures, such as pain, physical function, and disability.

#### 2.2.2. Exclusion Criteria

Exclusion criteria were listed as follows: title keywords unrelated to research topics; unclear articles; and incomplete study, study protocols, abstract, and review articles.

### 2.3. Research Tools: Critical Appraisal Instruments

The Physiotherapy Evidence Database (PEDro) scale was the critical appraisal instrument used in this study. The tool comprised eleven elements, and each element required a dichotomous yes/no response: 1 point was given to yes and 0 was allocated to no. The total score for the PEDro scale was 10. PEDro scores were excellent (9-10), good (6–8), fair (4-5), and poor (less than 4) [30].

### 2.4. Risk of Bias Assessment

The risks of bias within and across the study were assessed by using the Cochrane Effective Practice and Organization of Care risk of bias tool [32]. This tool comprised nine items, namely, random sequence generation, allocation concealment, similar baseline outcome, similar baseline characteristics, incomplete outcome data, blinding, contamination, selective outcome reporting, and other biases. All of the items were given a score of high risk, low risk, or unclear (Table 6).

### 2.5. Data Extraction

Data were extracted in terms of study design, sample size, inclusion and exclusion criteria, intervention, results, and conclusion (Table 2). The nature of intervention, outcome measures, and time point in each study were summarized (Tables 4 and 5).

### 2.6. Collection, Analysis, and Interpretation of Evidence

Evidence was collected by downloading all available full-text articles from online databases. Articles were then critically appraised by using the PEDro score. Articles were graded using the NHMRC Guideline Hierarchy Level of Evidence. PEDro scores were analyzed using SPSS version 20. Descriptive statistics was employed to determine the mean, median, minimum and maximum scores, standard deviation (SD), and variance between articles.

## 3. Results

### 3.1. Articles Supporting the Effectiveness of Exercise for Elderly People with LBP

Three articles were included [13, 15, 33]. Two of these included studies were RCTs [13, 33]. One article was an observational cohort study [31] (Table 2). Two articles were significantly different in terms of pain intensity related to exercise performed by the elderly people with LBP (Table 3). Hicks et al. [31] found that spinal extension, abdominal strengthening, and flexibility exercises improve the physical functions and pain severity of patients with LBP. Vincent et al. [28] also obtained significant results in terms of pain intensity reduction, disability, and pain catastrophizing after 4 months of resistance training.

### 3.2. Articles That Did Not Support the Evidence of Exercise Effectiveness for Elderly People with LBP

Vincent et al. [20] investigated the effect of total body resistance and lumbar extension exercise among obese elderly people with LBP. Although the back extensor is strengthened after exercise, pain intensity and back extensor strength do not significantly differ (*p* = 0.12).

**Figure 2:**
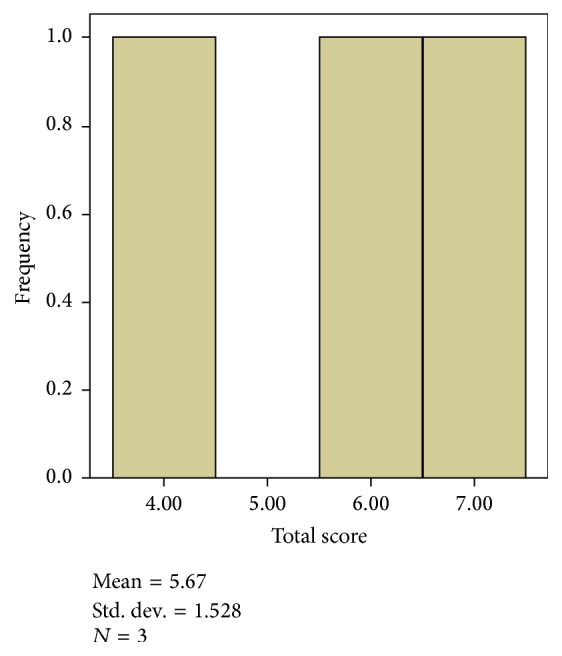
Histogram of total score of PEDro.

The mean of the three reviewed articles (*n* = 3) was 5.67 (Table 7). The quality of the studies on the basis of the mean of all articles was fair.  Figure 2 illustrates that each article scored 4, 6, and 7 out of the total PEDro score. The minimum and maximum scores were 3 and 7, respectively, with SD and variance of 2.33 and 1.528, respectively.

## 4. Discussion

### 4.1. Effects of Exercise on Elderly People with LBP

This study aimed to determine the reducing effects of strengthening exercises on the pain and symptoms experienced by the elderly people with LBP. To the best of our knowledge, this study is the first systematic review that evaluated the effectiveness of this exercise on elderly people. Three articles were reviewed, and the sample size was relatively small. One article comprised more than 50 subjects. One study did not include a control group [31]. The strengthening exercises were discussed in two studies, and one study described multicomponent exercises, including strengthening, stretching, and aerobic exercises. The selected studies were rated fair to good, with a generally low risk of bias.

Two of the three reviewed articles have reported that exercise reduces pain intensity after reassessment to a less degree than the baseline does [13, 14]. Vincent et al. [28] also found that pain catastrophizing is significantly alleviated in the TOTRX group that performs whole body resistance training, including lumbar extension exercise. Vincent et al. [28] also revealed findings consistent with those of Standaert et al. [33], who systematically reviewed the effect of exercise on the young age group with LBP. The authors summarized that exercise alleviates pain intensity and promotes functional improvement within 8 weeks. Kuss et al. [34] also performed a systematic review and suggested that physical therapy is associated with a slight-to-moderate decrease in pain intensity and improvement in functions of elderly people with chronic and nonspecific LBP. However, Kuss et al. [34] included studies on multidimensional physiotherapy approaches, such as mixed physiotherapy interventions of exercise, electrotherapy, and cognitive behavioral therapy. Therefore, the effectiveness of strengthening exercises for elderly people with LBP is difficult to prove because positive results may be contributed by other treatments rather than by exercise alone. By contrast, a standardized pain parameter known as numerical rating scale was used in our study; in this scale, the effect of pain reduction is likely valid [35].

The reviewed articles have suggested that exercise helps improve the functional performances of individuals with LBP after they complete the recommended exercise programs. Vincent et al. [20] found that the back extensor strength increases by approximately 20% in the intervention group. Vincent et al. [20] also found that exercise improves walking speed and endurance after 4 months of completing the strengthening exercise program. Although results do not reach statistical significance, this finding may suggest that strengthening exercises positively affect the functional performances of elderly people with LBP. Hicks et al. [31] reported a significant improvement in functional performance after 12 months of the exercise program. This finding is consistent with that of previous studies [17, 18], which revealed that exercise improves the mobility of elderly people with chronic pain. Both studies used multicomponent exercises and involved subjects with chronic pain and a mixed group of musculoskeletal pains. The mobility of the LBP group is possibly improved, although findings were unspecific to this group. Strengthening exercises also enhance the functional performances of elderly people with LBP.

Strengthening exercises also impede the possibility of disability in elderly people with LBP [13, 15]. Hicks et al. [31] identified the factors leading to the reduction of disability. One of these factors is adherence to the recommended exercise program. High adherence to exercise program is defined as a patient's participation in more than 75% of the exercise sessions; this phenomenon slightly contributes to the improvement of the disability level of the subjects. Therefore, adherence levels in each exercise program should be determined to ensure that pain and disability affecting elderly people with LBP are reduced. These findings are consistent with those of previous studies [19–22], which demonstrated that disability levels are decreased, although multidimensional approaches are used.

In summary, strengthening exercises are possibly effective for the improvement of the conditions of elderly people with LBP. Although aging weakens the muscle, muscle strength can be regained when appropriate exercise is administered to the affected elderly. As a result, progressive degenerative changes in muscles may be prevented and muscle functions and other functional performances of elderly people with LBP may be enhanced.

### 4.2. Methodological Consideration

This systematic review is limited by the lack of available articles on strengthening exercises within 10 years. Many available articles on strengthening exercises have combined their programs with other exercise types and multidimensional approaches. Current studies incorporate many interventions and multicomponent exercises. Although our systematic review summarizes the positive effect of strengthening exercises, our findings should be interpreted with caution because one of the reviewed articles involved multicomponent exercises, and exercise outcomes were unaffected by strengthening exercises alone.

In one of the reviewed articles, individuals were considered elderly when they are 50 years and above [31]. The classifications of older people vary worldwide; for instance, the World Health Organization defines elderly people on the basis of chronological age (65 years and above) or retirement age [36]. A study with this classification was included in our review because individuals in their country are classified as elderly when they reach 50 years and above.

Studies conducted in 2006 to 2016 investigated pain, functional performance, and disability. However, studies have rarely evaluated the effects of exercise on daily life activities, quality of life, and risk of falls. Hence, further experimental studies with high methodological quality should be performed. Different exercise types and other outcomes should also be compared to evaluate the benefits of each exercise and its effectiveness for elderly people with LBP.

## 5. Conclusion

Studies on the intervention of LBP in elderly people have recommended that strengthening exercises help alleviate pain and improve body functions. Therefore, physiotherapists should apply strengthening exercises as an effective treatment to improve physical functions and to prevent disability in clinical settings. Clinicians may also combine strengthening exercises with other exercise types and approaches, such as pain modalities and manual therapies, to further enhance the physical functions and reduce pain experienced by elderly people. Further studies on other types of back exercises, such as mobility, core stability, agility training, and balance exercises, should be conducted to investigate their benefits and efficacy in elderly people with LBP.

## Figures and Tables

**Table 1:** Example of literature searching using EBSCO EconLit 2006–2016 search strategy.

Search ID	Search terms	Result of article
1	Low back pain OR back pain OR lumbar pain OR back ache OR lumbago pain	25

2	Older people OR elderly OR older adult OR senior geriatric OR older population OR elders	6745

3	Strengthening Exercise OR resistance training	57

4	AND/1–3	16

**Table 2:** Data extractions.

Author	Study design	*N*	Exclusion	Inclusion	Intervention	Results	Conclusion
Vincent et al., 2014 [20]	RCT	*N* = 49	Being wheelchair bound, having a specific or an acute LBP injury, having spinal sternness with neurogenic claudication, having a back surgery within previous 2 years, or currently using any pharmacological or lifestyle weight loss intervention	Men and women aged 60–85, waist circumference of 88 (female) and 102 (male) and body mass index of ≥30 kg/m^2^, who suffer from LBP for ≥6 months and abdominal obesity and who are free from abnormal cardiovascular responses during a graded maximal walk test	Resistance exercise intervention (TOTRX): leg press, leg curl, leg extension, chest press, seated row, overhead press, triceps dips, lumbar extension, biceps curls, calf press, and abdominal curl Lumbar extension intervention (LEXT): lumbar extension Control group: no exercise	Gait speed increased Increase in walking endurance Increase in lumbar extension strength	The TOTRX and LEXT show improvement in patients walking endurance Lumbar extension strength shows moderate gain but is an important contributor to walking endurance in obese older adults with chronic low back pain

Hicks et al., 2012 [31]	Observational cohort study	*N* = 392	Unstable angina, uncontrolled hypertension, orthostatic hypotension, pulmonary disease requiring oxygen therapy, dementia, aphasia, back pain attributable to acute fracture, tumor, cancer, or infection, back or leg pain that worsened with spinal extension, and presence of 2 or more of the following signs of nerve root compression: diminished lower-extremity strength, sensation, or reflexes	Age 50 or greater with presence of back pain (thoracic or lumbar regions) for longer than 4 months, ability to rise from a chair and walk independently (with or without an assistive device), ability to travel to the exercise facility, and limited participation in physical activity at the initiation of the exercise program (<90 minutes of structured physical activity per week)	Exercise: spinal extension exercise, abdominal strengthening, and flexibility exercise	Improved back pain Improved adherence to exercise Improved functional performance Reduced disability	Older adults with back pain were able to safely participate in a community based exercise program with the majority reporting improved back pain status 12 months later

Vincent et al., 2014 [28]	RCT	*N* = 49	Wheelchair bound, resistance training regularly (participating in resistance exercise 3 or more times per week within the last 6 months), presence of specific low back pain due to an acute back injury such as a lumbar disc herniation or rupture, spinal stenosis with neurogenic claudication, back surgery within the previous two years, and the use of weight loss medication	Men and women 60.85 years of age, suffering from LBP for 6 months and abdominal obesity and free of abnormal cardiovascular responses during electrocardiogram (ECG) screening tests were eligible for the study	TOTRX: whole body resistance exercise + lumbar extension exercise Lumbar extension resistance exercise (LEXT): lumbar extension exercise Control group: (i) normal medical care and no exercise (ii) education on strengthening exercise, healthy nutritional choices, and back pain	Reduced pain Reduced disability Reduced pain catastrophizing	Total body resistance exercise (including lumbar extension exercise) was more effective than lumbar extension exercise alone in reducing self-reported disability scores due to back pain

**Table 3:** Trends in evidence.

References	Study design	Hierarchy level	PEDro score	Quality	Statistical precision	Tendency
Vincent et al., 2014 [20]	RCT	II	7/10	Good	CI = NA	±
					*p* > 0.05	

Hicks et al., 2012 [31]	Observational study	III-3	4/10	Fair	CI = 95%	Positive
					*p* < 0.006	

Vincent et al., 2014 [28]	RCT	II	6/10	Good	CI = NA	Positive
					*p* < 0.05	

CI: confidence intervals, NA: not applicable, positive: significant improvement found, negative: no improvement found, and ±: effect was found but not significant.

*p* value < 0.05 is significant.

**Table 4:** Summary of nature of intervention.

References	Intervention	Control group	Frequency
Vincent et al., 2014 [20]	Resistance exercise intervention (TOTRX): (i) leg press, leg curl, leg extension, chest press, seated row, overhead press, triceps dips, lumbar extension, biceps curls, calf press, and abdominal curl Lumbar extension intervention (LEXT): (i) lumbar extension	Yes: no exercise intervention with normal medical care and follow-up	TOTRX: 3 sessions per week for 4 months, one set of 15 reps of 60% 1 RM (i) progress with increase 2% of initial set per week for four months LEXT: 2 sets of lumbar exercise, 15 reps each, 3 times per week for four months

Hicks et al., 2012 [31]	Strengthening: (i) abdominal strengthening, thoracolumbar, and scapula retraction in sitting or standing position or lying Stretching: (i) hamstring and calf Endurance: (i) 5–10 minutes walking	No	12 months, one hour, twice weekly, per session (20–30-time reps)

Vincent et al., 2014 [28]	Resistance exercise intervention (TOTRX): leg press, leg curl, leg extension, chest press, seated row, overhead press, triceps dip, lumbar extension, biceps curl, calf press, abdominal curl, and the lumbar extension exercise, one set of 15 reps. Lumbar extension intervention LEXT: lumbar extension exercise 15 reps × 2 sets	Yes: education on strengthening exercise, healthy nutritional choices, and back pain	TOTRX: 15 reps, one set, 60% of 1 RM, 3 times per week for 4 months

**Table 5:** Outcome measure and time point.

References	Outcome measure	Statistical tests	Time point
Vincent et al., 2014 [20]	Low back pain severity score Numerical rating scale Maximal low back and leg strength Walking endurance and gait speed	Chi-square test Univariate analyses of variance	4 months

Hicks et al., 2012 [31]	Numerical rating scale Geriatric Depression Scale (GDS) The Short Physical Performance Battery (SPPB) including three-stance balance test, 4-meter gait speed test, and a five-repetition chair-stand test Roland Morris Back Pain Disability Questionnaire	Descriptive Statistics Multivariable logistic regression analyses	12 months

Vincent et al., 2014 [28]	Numerical rating scale Modified Oswestry Disability Index (mODI) Roland Morris Disability Questionnaire Psychological assessment: (i) Tampa Scale of Kinesiophobia, (ii) Fear-Avoidance Beliefs Questionnaire (iii) Pain Catastrophizing Scale	Kruskal-Wallis Repeated measures ANOVA	4 months

**Table 6:** Appraisal of risk of bias, according to Cochrane Effective Practice and Organization of Care risk of bias tool.

Criterion/articles	Random sequence generation	Allocation concealment	Similar baseline outcome	Similar baseline characteristics	Incomplete outcome data	Blinding	Contamination	Selective outcome reporting	Other bias
Vincent et al., 2014 [20]	Unclear	Low	Low	Low	Low	Low	Low	Unclear	Unclear
Hicks et al., 2012 [31]	Unclear	Unclear	Low	Low	High	High	Unclear	Low	Unclear
Vincent et al., 2014 [28]	Low	Low	Low	Low	Low	Low	Low	Low	Unclear

**Table 7:** Overall findings of strength of evidence (descriptive statistics).

*N* (number of articles)	3
Mean	5.67
95% confidence interval for mean	
Lower bound	1.87
Upper bound	9.46
Median	6
Standard deviation	2.33
Variance	1.528
Minimum	4
Maximum	7
